# Metabolic Profiling Reveals Insights Into Bladder Cancer Pathogenesis and Recurrence

**DOI:** 10.1155/aiu/6524384

**Published:** 2026-03-30

**Authors:** Hüseyin Saygın, Serkan Bolat, Demet Kablan, Hayrettin Yavuz, Meltem Kurt Yenihan, Adem Kır, Abuzer Öztürk, Akif Doğan, Onur Şenol, Halef Okan Doğan, Esat Korğalı

**Affiliations:** ^1^ Department of Urology, Sivas Cumhuriyet University, Sivas, Turkey, cumhuriyet.edu.tr; ^2^ Department of Biochemistry, Sivas Cumhuriyet University, Sivas, Turkey, cumhuriyet.edu.tr; ^3^ Department of Pediatrics, University of Virginia, Charlottesville, Virginia, USA, virginia.edu; ^4^ Department of Analytical Chemistry, Atatürk University, Erzurum, Turkey, atauni.edu.tr

**Keywords:** (±)12-HETE, arachidonic acid, bladder cancer, metabolomics, PGE2, steroid hormones

## Abstract

Bladder cancer is characterized by abnormal cell proliferation within the bladder lining, yet the molecular mechanisms underlying its pathogenesis remain incompletely understood. This study aimed to identify metabolic differences between bladder cancer patients and healthy controls, as well as between patients with and without cancer recurrence, to elucidate the molecular mechanisms underlying disease progression and recurrence. A total of 102 participants were enrolled, comprising 82 individuals diagnosed with bladder cancer and 20 healthy controls. Based on cystoscopy and pathology findings, bladder cancer patients were further categorized into two groups: 29 with tumor recurrence and 41 without recurrence. Urinary metabolic profiling was conducted using ultra‐performance liquid chromatography coupled with quadrupole time‐of‐flight mass spectrometry (UPLC/Q‐TOF/MS). Data acquisition, classification, and metabolite identification were performed using Mass Profiler Professional and the XCMS online platform (https://xcmsonline.scripps.edu). Compared to healthy controls, patients exhibited downregulation of 11‐hydroxyandrosterone and prostaglandin E2 (PGE2), while (±)12‐hydroxyeicosatetraenoic acid ([±]12‐HETE), 5α‐dihydrodeoxycorticosterone, and 21‐hydroxypregnenolone were upregulated. Notably, 11‐hydroxyandrosterone was further downregulated in patients with recurrent disease compared to those without recurrence. The highest area under the curve (AUC) values for distinguishing bladder cancer and recurrence were observed for (±)12‐HETE and PGE2. Elucidating the roles of steroid hormones and arachidonic acid metabolism in bladder cancer may provide critical insights into its molecular mechanisms and facilitate the development of novel therapeutic and diagnostic strategies to improve outcomes for patients.

**Highlights**
• Significant differences in metabolite levels were observed between patients with bladder cancer and healthy controls.
• Downregulation of 11‐hydroxyandrosterone in recurrent bladder cancer patients suggests its potential as a marker for recurrence.
• (±)12‐HETE and PGE2 showed high discriminatory power in differentiating bladder cancer from healthy individuals.
• Understanding the roles of steroid hormones and arachidonic acid provides avenues for targeted therapies and diagnostics.

## 1. Introduction

Bladder cancer is recognized as a significant global health problem, with more than 600,000 new cases and approximately 220,000 deaths reported each year worldwide [1]. Its incidence increases with age and is generally more common in individuals over 55 years of age [2]. Established risk factors include smoking, chemical exposures, age, gender, and genetic predisposition. Early detection and successful treatment have the potential to halt disease progression and improve patient outcomes [3]. However, the diagnosis and management of bladder cancer present numerous challenges. In the early stages, symptoms are often vague or absent, leading to delayed detection and limited treatment options. Current standard diagnostic and monitoring methods include cystoscopy, urine cytology, and imaging, but each has significant limitations [4, 5]. Cystoscopy, while considered the gold standard, is invasive, costly, and can cause considerable physical and psychological discomfort; moreover, it may miss small carcinoma in situ (CIS) lesions and requires lifelong surveillance in nonmuscle‐invasive bladder cancer (NMIBC). Urine cytology, although noninvasive, suffers from low sensitivity and specificity, particularly for low‐grade tumors with a substantial risk of false‐negative results [5–7]. These drawbacks highlight the urgent need for more sensitive, specific, and noninvasive diagnostic approaches to enable earlier detection and more effective monitoring of bladder cancer. Despite the identification of several potential biomarkers in previous research, none have yet been implemented in routine clinical practice [8–12]. Thus, there is a critical need for more sensitive and specific diagnostic tools to improve early detection and disease management.

Untargeted metabolomics is an analytical approach that enables the comprehensive identification and characterization of metabolites in biological samples without reliance on predefined target lists. This methodology produces a comprehensive metabolic profile, enhances understanding of biological processes and disease mechanisms, and supports the discovery of novel biomarkers. Untargeted metabolomics is therefore a promising strategy for identifying biomarkers and metabolic pathways implicated in cancer development, progression, and treatment response [13]. Despite its potential, few studies have applied untargeted metabolomics to bladder cancer, particularly regarding disease recurrence [10, 14, 15]. Characterizing metabolic alterations in bladder cancer may facilitate the identification of new biomarkers for early diagnosis and prognosis, including recurrence, thereby advancing early detection, personalized therapy, and improved clinical outcomes.

This study represents the first untargeted ultra‐performance liquid chromatography coupled with quadrupole time‐of‐flight mass spectrometry (UPLC/Q‐TOF/MS)–based urinary metabolomics investigation in a Turkish bladder cancer population, addressing a significant gap in global bladder cancer metabolomics datasets. The research employed a dual‐group comparison: (i) bladder cancer patients versus healthy controls to identify potential diagnostic biomarkers and (ii) recurrence‐positive versus recurrence‐negative bladder cancer patients to identify prognostic biomarkers. Data acquisition was conducted in both positive and negative ion modes to ensure broad coverage of metabolites and a comprehensive metabolic profile. The primary objectives were to characterize urinary metabolic alterations in bladder cancer, elucidate the molecular mechanisms underlying disease progression, and identify candidate biomarkers for early detection and prediction of recurrence.

## 2. Materials and Methods

### 2.1. Study Population and Sample Collection

This prospective study was conducted at the Department of Urology and the Department of Clinical Biochemistry, Sivas Cumhuriyet University School of Medicine. A total of 102 participants were enrolled, including 82 patients diagnosed with bladder cancer and 20 healthy controls. Male participants included in the control group were required to meet the following predefined inclusion criteria: an age of under 60 years, a nonsmoking status, and no previous diagnosis of bladder cancer. Furthermore, participants had not received any treatment related to bladder cancer. Exclusion criteria comprised a history of other malignancies requiring therapeutic intervention, active infections, and ongoing medical treatment. After comprehensive clinical and laboratory evaluations, all bladder cancer patients underwent transurethral resection of bladder tumor (TURBT). An early single dose of gemcitabine chemotherapy was administered within 24 h postsurgery, unless contraindicated. Twelve patients were excluded from the analysis due to the presence of muscle‐invasive bladder tumors, in accordance with the study’s inclusion criteria. Urine samples were collected at the time of diagnosis, prior to cystoscopy. Based on cystoscopy and pathology findings, patients were categorized into two groups: 29 with tumor recurrence and 41 without. The mean age of the patients was 66 years. Within the study population, 66 patients were identified as smokers. In 45 cases, the tumor size exceeded 3 cm. Multicentric disease was observed in 39 patients, and high‐grade tumors were identified in 39 patients. In 38 cases, the presence of lamina propria invasion was identified. The study complied with national regulations, institutional policies, and the principles of the Declaration of Helsinki for research involving human subjects. Ethical approval was obtained from the Institutional Review Board of Sivas Cumhuriyet University (approval number: 2020‐02/03). Written informed consent was obtained from all participants. Table 1 summarizes the clinical and demographic characteristics of the study population.

**TABLE 1:** Clinical and demographic variables in study population.

Variables	Bladder cancer (*n* = 70)		Control (*n* = 20)	Total (*n* = 90)
	Recurrence (+) (*n* = 29)	Recurrence (−) (*n* = 41)		
Age (years)	63 ± 12	67 ± 11	63 ± 13	66 ± 12
Patients (male/female)	26/3	33/8	17/3	76/14
Smoking	25 (86%)	28 (68%)	15 (75%)	68 (76%)
Tumor Size (≤ 3/> 3 cm)	11/18	14/27	—	25/45
Multicentric (−/+)	12/17	27/14	—	39/31
Tumor grade (High/Low)	15/14	16/25	—	31/39
Gemcitabine therapy	27	38	—	65
Tumor Stage (pTa, pT1)	13/16	19/22	—	32/38

### 2.2. Sample Preparation

Fasting morning urine samples were collected from all participants and centrifuged at 3500 rpm at 4°C for 15 min. The resulting supernatant was aliquoted and immediately frozen at −80°C for later analysis. For sample preparation, 100 μL of thawed urine was mixed with 200 μL of an acetonitrile:methanol (1:1, v/v) solution. The mixture was vortexed and incubated at −20°C for 60 min. Subsequently, the samples were centrifuged at 13,000 rpm at 4°C for 15 min. The supernatant was then dried for 12 h using a vacuum concentrator (Thermo Fisher Scientific, Waltham, MA, USA). The dried extracts were reconstituted in 100 μL of acetonitrile:methanol (1:1, v/v). The reconstituted samples were centrifuged again at 13,000 rpm and 4°C for 15 min to remove any remaining insoluble debris.

### 2.3. Quality Control (QC)

A strict QC process was applied throughout the analysis to ensure reliability and reproducibility. To ensure mass accuracy, a reference solution was continuously infused into the source for internal calibration throughout the analysis. For this purpose, purine (m/z 121.0508) and hexakis phosphazinen (m/z 922.0097) were used as reference ions. Before sample analysis, LC–MS system suitability was verified by repeated injections of pooled QC samples to confirm stable chromatographic performance, retention time stability, signal reproducibility, and mass accuracy. A pool of representative biological samples was prepared and injected at regular intervals throughout the analytical run as QC material. Pooled QC samples were injected every nine runs to monitor system stability, with five initial QC injections used for system conditioning and excluded from data analysis. This allowed for the assessment of system stability, batch effects, and overall data quality.

**FIGURE 1:**
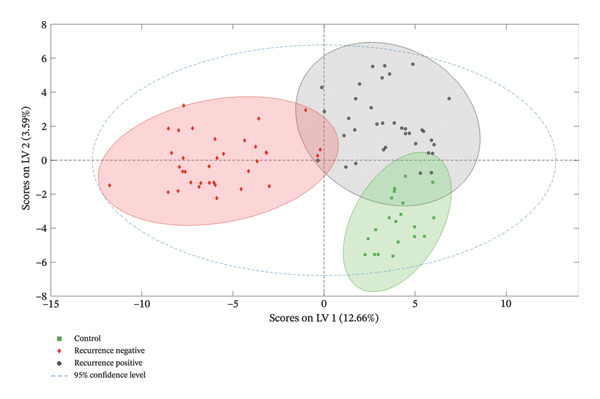
PCA score plot of latent variables in accordance with the partial least squares discriminant analysis (PLS‐DA) model demonstrating a clear separation among the three groups, indicating distinct metabolic profiles. LV1 and LV2 explained 12.66% and 3.59% of the variance, respectively, with 95% confidence ellipses confirming robust group separation and indicating distinct metabolic phenotypes linked to disease presence and recurrence status.

QC performance was evaluated based on signal reproducibility, retention time stability, and mass accuracy. Feature reproducibility was assessed by calculating the relative standard deviation (%RSD) of peak intensities across QC injections, and features with %RSD > 30% were excluded from further analysis. Features showing unstable retention times were also removed. In addition, only features detected in ≥ 80% of QC injections and in ≥ 70–80% of samples within at least one biological group were retained for further analysis. Multivariate analysis (principal component analysis [PCA]) was used to evaluate QC clustering behavior, confirming analytical stability across the batch. QC‐based signal correction and normalization were applied prior to statistical analysis to reduce analytical variation and batch effects (Figure 1).

In addition to the QC procedures, the use of blank samples (acetonitrile/methanol 1/1 [v/v]) was a part of the QC strategy to assess the presence of any background noise or contaminants originating from the sample preparation or analysis process. QCs and blank samples were injected at the beginning of every nine samples. We used these blanks to filter our dataset: features that were present in the procedural blanks with intensities comparable to those in the biological samples were considered contaminants and excluded from the final feature list.

### 2.4. Untargeted Analysis via Q‐TOF MS

The UPLC/Q‐TOF/MS system used in this study consisted of an Agilent 1290 Infinity LC system coupled with an Agilent 6530 Accurate‐Mass Q‐TOF MS, both manufactured by Agilent in the USA. The chromatographic separation was performed using a ZORBAX RRHD Eclipse Plus C18 column with dimensions of 2.1 × 100 mm and a particle size of 1.8 μm, also from Agilent. For the mobile phase system, a gradient elution method was employed. The mobile phase consisted of 0.1% formic acid in water (A) and acetonitrile (B). The elution gradient was as follows: 0–0.5 min, 2% B; 0.5–4 min, 20% B; 4–8 min, 50% B; 8–9 min, 95% B; 9–9.25 min, 2% B; and 9.25–15 min, 2% B for column equilibration. The flow rate of the mobile phase was maintained at 0.6 mL/min. The column temperature was set at 55°C throughout the analysis. The injection volume was 4.0 μL. The MS was operated in full scan mode. The ionization source capillary voltage was set to 3.5 kV in positive and negative scanning modes. The nebulizer gas pressure was set to 45 psi. The dry gas temperature was set to 250°C. The collision energy was stepped from 21 to 45 eV. The mass detection range was 50–1500 in positive mode and 65–1500 in negative mode. The acquisition rate was 1.5 spectra/s.

### 2.5. Extraction of Raw Data and Putative Metabolite Annotation

The raw data were acquired using Agilent MassHunter Data Acquisition Workstation software (Agilent Technologies, USA) in “.d” format. The raw data were processed using MassHunter Profinder 10.0.2 software and converted to “.cef” format. The converted files were uploaded into Mass Profiler Professional (MPP) 15.1.1 software for normalization, feature extraction, alignment, and putative metabolite annotation.

Metabolite annotation was performed by matching the accurate mass and retention time of observed peaks to metabolite databases using ±15 ppm mass tolerance and ±0.3 min retention time tolerance. MS/MS fragmentation patterns were further compared with publicly available spectral databases to support annotation confidence.

Each experimental m/z was searched against METLIN, LIPID MAPS Structure Database (LMSD), and the Human Metabolome Database (HMDB). As no authentic reference standards were used for confirmation, all metabolite annotations were considered putative identifications corresponding to Metabolomics Standards Initiative (MSI) level 2 (putatively annotated compounds), according to the MSI reporting guidelines [16].

### 2.6. Statistical Analysis

Statistically significant metabolic features were extracted from the metabolite intensity table using fold changes and *p* values obtained from univariate statistical testing (Student’s *t*‐test or Mann–Whitney U test, as appropriate). Metabolic features with a fold change of ≥ 1.5 and an adjusted *p* value (false discovery rate [FDR]‐corrected) of < 0.05 were considered statistically significant and compiled into a list of significant features. Multiple testing correction was performed using the Benjamini–Hochberg FDR method. A significant feature was excluded from the list if it was also found in the blank method. Receiver operating characteristic (ROC) curves were plotted for the metabolites to detect bladder cancer and recurrence conditions. The area under the curve (AUC) values, sensitivity, specificity, and likelihood ratios (LRs) were calculated. All statistical analyses and ROC curve analyses were performed using GraphPad Prism (version X.X, GraphPad Software, San Diego, CA, USA).

## 3. Results

PCA was performed using the MPP 15.1.1 software to explore the metabolic differences between controls, recurrence‐negative, and recurrence‐positive bladder cancer patients. The PCA score plot (Figure 1) demonstrated a clear separation among the three groups, indicating distinct metabolic profiles. The first two latent variables (LV1 and LV2) explained 12.66% and 3.59% of the variance, respectively, with the 95% confidence ellipses confirming the robustness of the group discrimination. This finding indicates that a substantial portion of the metabolic variation among the samples can be attributed to differences between the control, recurrence‐negative, and recurrence‐positive groups, suggesting distinct underlying metabolic phenotypes associated with disease presence and recurrence status.

**FIGURE 2:**
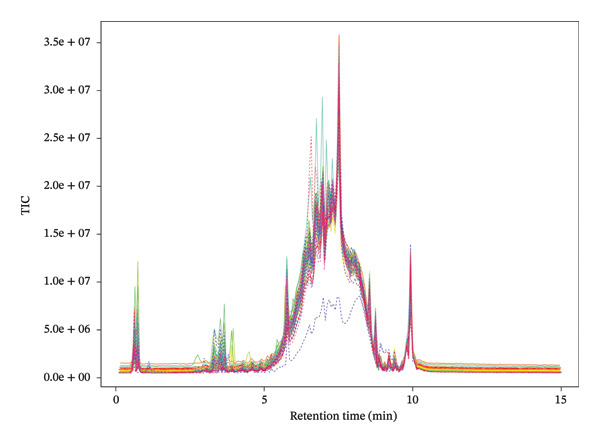
Total ion chromatogram (TIC) of each sample via ultra‐performance liquid chromatography coupled with quadrupole time‐of‐flight mass spectrometry (UPLC/Q‐TOF/MS). A total of 272 features were detected in the positive ion mode and 178 features in the negative ion mode. Of these, 37 metabolites in negative ion mode and 14 metabolites in positive ion mode were successfully annotated based on accurate mass matching to KEGG and HMDB databases.

The total ion chromatograms (TICs) obtained from both positive and negative ion modes are shown in Figure 2. A total of 272 features were detected in the positive ion mode and 178 features in the negative ion mode. Of these, 37 metabolites in the negative ion mode and 14 metabolites in the positive ion mode were successfully annotated based on accurate mass matching with the KEGG and HMDB databases.

**FIGURE 3:**
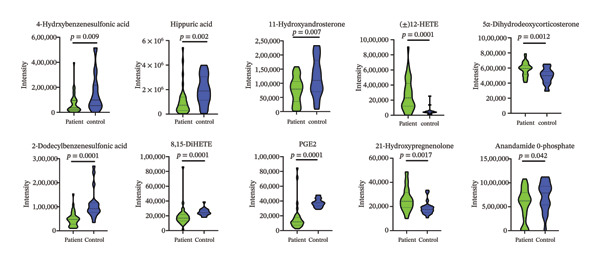
Violin plots illustrating the distribution of urinary metabolite intensities in bladder cancer patients compared to healthy individuals. Each plot shows the relative intensity, median, and variability for the respective metabolites in both groups, along with *p* values indicating statistical significance. Bladder cancer patients (*n* = 70; recurrence‐positive, *n* = 29; recurrence‐negative, *n* = 41) and healthy controls (*n* = 20). (±)12‐HETE; (±)12‐hydroxyeicosatetraenoic acid, 8,15‐DiHETE; 8,15‐dihydroxyicosatetraenoic acid, PGE2; prostaglandin E2.

Comparative statistical analysis revealed several metabolites with significant differences between patient (*n* = 70) and healthy control (*n* = 20) groups (Table 2). Notably, (±)12‐Hydroxyeicosatetraenoic acid [(±)12‐HETE], prostaglandin E2 (PGE2), and 2‐dodecylbenzenesulfonic acid exhibited the largest fold changes. Among these, (±)12‐HETE showed a marked upregulation (fold change: 5.42, *p* < 0.0001), while PGE2 demonstrated a significant downregulation (fold change: 3.36, *p* < 0.0001). Other altered metabolites included 4‐hydroxybenzenesulfonic acid, hippuric acid, 11‐hydroxyandrosterone, 8,15‐dihydroxyicosatetraenoic acid (8,15‐DiHETE), anandamide O‐phosphate, 5α‐dihydrodeoxycorticosterone, and 21‐hydroxypregnenolone. Violin plots of these significantly different metabolites (Figure 3) illustrate the distribution, median, and variability in intensity values between patients and controls, with corresponding *p* values indicating statistical significance.

**TABLE 2:** List of significantly different metabolites between groups.

Groups	Compound	KEGG&HMDB ID	Ion polarity	Measured mass	Exact mass	Reference mass difference	Retention time	Fold	Adj‐*p*value	Regulation
Patient vs Control	4‐Hydroxybenzenesulfonic acid	C14274	Negative	236.1434	236.1412	2.16	9.62	2.77	0.009	Down
	Hippuric acid	C01586	Negative	179.0582	179.0582	−0.04	3.53	2.60	0.002	Down
	11‐Hydroxyandrosterone	C14606	Negative	306.2213	306.2194	6.19	9.83	1.45	0.007	Down
	(±)12‐HETE	C14777	Negative	320.2362	320.2351	−0.01	9.42	5.42	< 0.0001	Up
	2‐Dodecylbenzenesulfonic acid	HMDB31031	Negative	326.1908	326.1915	−0.88	9.54	1.94	< 0.0001	Down
	8,15‐DiHETE	C17770	Negative	336.2309	336.2301	0.85	9.21	1.43	< 0.0001	Down
	PGE2	C00584	Negative	352.2265	352.2249	1.60	9.57	3.36	< 0.0001	Down
	Anandamide 0‐phosphate	C19913	Positive	427.2479	427,2487	−0.85	4.58	1.26	0.042	Down
	5α‐Dihydrodeoxycorticosterone	C18040	Positive	332.2337	332.2351	0.29	3.09	1.14	0.0012	Up
	21‐Hydroxypregnenolone	C05485	Positive	332.2337	332.2351	0.29	3.09	0.13	0.0017	Up

Recurrence positive vs recurrence negative	p‐Cresol sulfate	C06677	Negative	188.0137	188.0143	−0.56				Up
	11‐Hydroxyandrosterone	C14606	Negative	306.2213	306.2194	6.19	9.83	9.56	< 0.0001	Down

*Note:* (±)12‐HETE: 12(S)‐hydroxy‐(5Z,8Z,10E,14Z)‐eicosatetraenoic acid, 8,15‐DiHETE: 8,15‐dihydroxyeicosa‐5,9,11,13‐tetraenoic acid, PGE2: prostaglandin E2.

**FIGURE 4:**
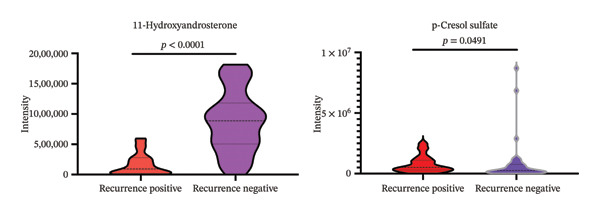
Violin plots illustrating the distribution of urinary metabolite intensities in recurrence‐positive (*n* = 29) and recurrence‐negative (*n* = 41) bladder cancer patients. Each plot shows the relative intensity, median, and variability for the respective metabolite, with *p* values indicating the statistical significance of group differences.

Further analysis of recurrence‐positive versus recurrence‐negative bladder cancer patients identified two metabolites with significant discriminatory power: 11‐hydroxyandrosterone and p‐cresol sulfate (Table 2). Violin plots show markedly lower levels of 11‐hydroxyandrosterone in recurrence‐positive patients (*p* < 0.0001) and higher levels of p‐cresol sulfate (*p* = 0.0491) compared to recurrence‐negative patients (Figure 4).

**FIGURE 5:**
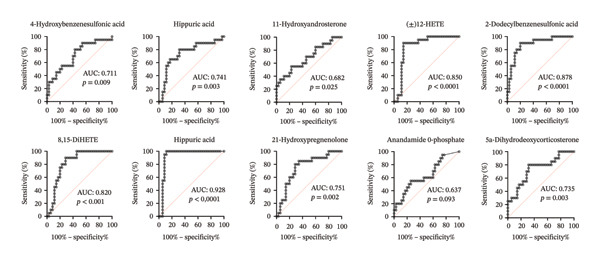
Evaluation of the diagnostic performance of metabolites predicting bladder cancer using receiver operating characteristic (ROC) curve analysis. PGE2 achieved the highest AUC value of 0.928 (95% CI: 0.848–1.00, *p* < 0.0001), indicating excellent discriminative ability. (±)12‐HETE; (±)12‐hydroxyeicosatetraenoic acid, 8,15‐DiHETE; 8,15‐dihydroxyicosatetraenoic acid, PGE2; prostaglandin E2.

**FIGURE 6:**
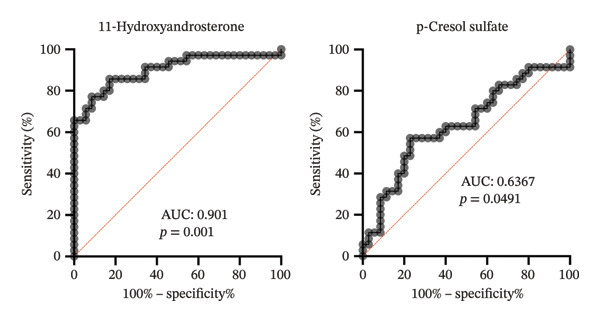
Evaluation of the diagnostic performance of metabolites predicting recurrence using receiver operating characteristic (ROC) curve analysis. 11‐hydroxyandrosterone demonstrated the highest accuracy with an AUC of 0.901 (95% CI: 0.8255–0.9770, *p* = 0.001), followed by p‐cresol sulfate (AUC: 0.6367, *p* = 0.0491).

ROC curve analysis was conducted to evaluate the diagnostic performance of each metabolite (Figures 5 and 6). In the comparison between patients and controls, PGE2 achieved the highest AUC value of 0.928 (95% CI: 0.848–1.00, *p* < 0.0001), indicating excellent discriminative ability. Other metabolites with high diagnostic performance included 2‐dodecylbenzenesulfonic acid (AUC: 0.878), (±)12‐HETE (AUC: 0.850), and 8,15‐DiHETE (AUC: 0.820) (Table 3 and Figure 5). For recurrence prediction, 11‐hydroxyandrosterone exhibited strong prognostic performance for predicting recurrence, with an AUC of 0.90 (95% CI: 0.82–0.97, *p* < 0.0001), a sensitivity of 91.43%, and a specificity of 65.71%, yielding a LR of 2.66. In contrast, p‐cresol sulfate showed limited predictive value, with an AUC of 0.57 (95% CI: 0.40–0.72, *p* = 0.0491), sensitivity of 57.14%, specificity of 77.14%, and a LR of 2.50 (Figure 6).

**TABLE 3:** ROC analysis results indicating the prognostic performances of metabolites in predicting bladder cancer.

Metabolites	ROC statistic		Diagnostic statistic		
	AUC (95% CI)	*p* value	Sensitivity	Specificity	LR
4‐Hydroxybenzenesulfonic acid > 28,512	0.71 (0.56–0.85)	**0.009**	90 (69.90–98.22)	45.71 (30.47–61.81)	1.65
Hippuric acid > 513,499	0.74 (0.59–0.88)	**0.003**	82.35 (69.90–98.22)	94.06 (27.98–59.14)	1.57
11‐Hydroxyandrosterone > 69,106	0.80 (0.58–0.91)	**0.025**	80.00 (58.40–91.93)	40 (25.55–56.43)	1.33
(±)12‐HETE < 72,638	0.85 (0.74–0.95)	**<** **0.001**	90.00 (69.90–98.22)	85.71 (70.62–93.74)	6.3
2‐Dodecylbenzenesulfonic acid > 61,343	0.87 (0.78–0.97)	**<** **0.001**	90.00 (64.11–89.96)	80.00 (64.11–89.96)	4.5
8,15‐DiHETE > 19,890	0.82 (0.70–0.93)	**<** **0.0001**	90.00 (69.90–98.22)	71.43 (54.95–83.67)	3.15
PGE2 > 25,189	0.92 (0.83–1.00)	**<** **0.001**	94.12 (83.18–100.0)	85.15 (72.67–95.18)	8.25
21‐Hydroxypregnenolone < 200,530	0.75 (0.61–0.88)	**0.002**	0.80 (58.40–91.93)	71.43 (54.95–83.67)	2.8
Anandamide 0‐phosphate > 496,208	0.63 (0.4–0.79)	**0.093**	80.00 (58.40–91.93)	37.14 (23.17–53.66)	1.27
5α‐Dihydrodeoxycorticosterone < 277,016	0.73 (0.59–0.87)	**0.003**	80.00 (58.40–91.93)	68.57 (52.02–81.45)	2.545

*Note:* All the bold *p* values are significant except for the *p* value for Anandamide 0‐phosphate which is 0.093.

Abbreviations: AUC, area under the curve; CI, confidence interval; LR, likelihood ratio; ROC, receiver operating characteristics.

## 4. Discussion

(±)12‐HETE is a bioactive lipid derived from arachidonic acid via the 12‐lipoxygenase (12‐LOX) pathway, playing a significant role in cancer biology. It has been shown to contribute to various cancer hallmarks, including cell proliferation, angiogenesis, metastasis, and evasion of apoptosis [17–22].

Although elevated levels of (±)12‐HETE have been observed in various cell culture models of different cancers, such as gastric [20] and ovarian cancers [21], highlighting its role as a potent oncogenic factor, only a limited number of studies have evaluated the levels of this metabolite in cancer patients. In these studies, higher levels of (±)12‐HETE were reported in patients with lung cancer [16] and breast cancer [23]. We detected higher levels of (±)12‐HETE in patients compared to the healthy controls. Although these findings suggest that (±)12‐HETE upregulation may contribute to tumor biology, it remains unclear whether this elevation plays a causal role in cancer initiation and progression or simply reflects an epiphenomenon of malignant transformation. Some experimental studies have indicated that pharmacological inhibition of 12‐LOX can attenuate tumor growth, angiogenesis, and metastasis, supporting the concept of a functional role. However, these data are largely derived from preclinical models, and translation to human cancer therapy requires caution. Therefore, while targeting (±)12‐HETE metabolism may represent a promising therapeutic avenue, current evidence is insufficient to conclude that suppressing elevated (±)12‐HETE levels would definitively improve clinical outcomes. Future studies should aim to clarify the mechanistic contribution of (±)12‐HETE to bladder cancer pathogenesis and assess whether its inhibition yields tangible therapeutic benefits in patients.

In the present study, we observed elevated levels of 21‐hydroxypregnenolone and 5α‐dihydrodeoxycorticosterone, along with decreased levels of 11‐hydroxyandrosterone, in bladder cancer patients compared to healthy controls. Notably, 11‐hydroxyandrosterone showed the highest AUC value for predicting recurrence, highlighting its potential clinical relevance. These metabolites are intermediates in steroid hormone biosynthesis, a pathway increasingly recognized for its involvement in tumor biology through modulation of androgen receptor (AR), glucocorticoid receptor (GR), and mineralocorticoid receptor (MR) signaling [24]. 5α‐Dihydrodeoxycorticosterone, a 21‐hydroxysteroid derived from deoxycorticosterone, has been implicated in AR activation in castration‐resistant prostate cancer, suggesting that it may act as a ligand or modulator of AR signaling [25]. In bladder cancer, AR expression has been demonstrated in tumor tissue, and AR positivity is associated with more aggressive disease phenotypes [26, 27].

Through AR activation, these metabolites may promote transcriptional programs that enhance urothelial cell proliferation, inhibit apoptosis, and facilitate epithelial–mesenchymal transition (EMT), thereby contributing to tumor progression and possibly recurrence. The observed reduction in 11‐hydroxyandrosterone, an androgen catabolite, may reflect altered androgen metabolism favoring the accumulation of active or precursor steroids, thus sustaining AR‐driven oncogenic signaling. Its association with recurrence could indicate that patients with persistently low levels have a more active androgen signaling axis, which may support tumor persistence or regrowth after initial treatment. Beyond direct effects on tumor cells, dysregulation of steroid hormone metabolism can influence the tumor microenvironment. Steroid hormones modulate immune cell infiltration, cytokine secretion, and angiogenesis, potentially creating an immunosuppressive niche favorable for tumor survival [28–30]. The concurrent alterations in multiple steroid pathway intermediates observed in our study suggest a broader reprogramming of steroid metabolism in bladder cancer. Taken together, these findings support the hypothesis that steroid hormone metabolism, particularly AR‐related pathways, plays a functional role in the biology of bladder cancer. Understanding the interplay between steroid metabolites, hormone receptor signaling, and the tumor immune microenvironment could open new avenues for therapeutic intervention. Moreover, the strong predictive value of 11‐hydroxyandrosterone for recurrence highlights its promise as a noninvasive biomarker for disease monitoring and personalized treatment planning.

Hippuric acid is a typical microbiome–host co‐metabolite, generated through the conjugation of benzoate—produced by gut microbial metabolism of dietary polyphenols and aromatic amino acids—with glycine in the liver [31–33]. Circulating hippurate levels are positively associated with microbial diversity, microbial gene richness, and overall metabolic health. Reduced hippurate concentrations in bladder cancer patients, as observed in this study, may reflect gut microbiota dysbiosis leading to decreased microbial benzoate production, an impaired hepatic glycine conjugation capacity, or metabolic reprogramming associated with malignancy. Previous studies have demonstrated that hippurate levels correlate with benzoate biosynthesis modules and that supplementation with hippurate can improve glucose homeostasis in experimental models [34]. These findings suggest that reduced hippurate is not merely a passive marker of altered metabolism but may also contribute to disease‐associated metabolic dysfunction.

P‐cresol sulfate is a protein‐bound uremic toxin generated from microbial fermentation of tyrosine and phenylalanine to p‐cresol, followed by sulfation in the host. It is primarily derived from the colonic microbiota, and its circulating concentration is influenced by renal clearance and the efficiency of tubular secretion. P‐cresol sulfate has been shown to exert pro‐oxidant, pro‐inflammatory, and endothelial‐damaging effects, as well as to modulate immune responses [35–38]. Experimental studies have reported that p‐cresol and its conjugates can promote proliferation and migration of certain cancer cell types, possibly through signaling pathways involving miR‐21 and HIF‐1α [39]. In our cohort, recurrence‐positive patients exhibited significantly higher p‐cresol sulfate levels, which may indicate a persistent proinflammatory and immunomodulatory tumor microenvironment driven by microbiota‐derived metabolites or differences in renal excretion dynamics related to transporter activity. This association supports the potential role of p‐cresol sulfate as a biomarker of recurrence risk in bladder cancer.

2‐Dodecylbenzenesulfonic acid belongs to the class of linear alkylbenzene sulfonates (LAS), a common surfactant component in detergents and industrial cleaning products (HMDB0031031). The presence of such compounds in human metabolomic profiles is often considered a marker of environmental exposure rather than an endogenous metabolic process. In clinical samples, alkylbenzene sulfonates have been associated with differences in lifestyle, occupational exposure, and environmental contact, as well as with markers of inflammation [40, 41]. In our study, 2‐DBSA levels were decreased in bladder cancer patients compared with controls. This finding may reflect altered environmental exposure patterns, differences in xenobiotic metabolism, or changes in renal clearance, rather than a tumor‐specific metabolic pathway.

Dysregulation of the steroid hormone pathway has been implicated in the pathogenesis of certain cancers [42]. The interplay between steroid hormones and immune cell interactions may also influence cancer progression [43]. Although the precise mechanisms by which these metabolites contribute to cancer remain to be fully elucidated, their capacity to modulate key steroid hormone signaling pathways and cellular processes involved in oncogenesis warrants further investigation. A deeper understanding of the steroid hormone pathway in bladder cancer may inform the development of novel therapeutic strategies targeting these signaling pathways. Furthermore, 11‐hydroxyandrosterone may serve as a diagnostic marker for assessing the risk of recurrence.

PGE2 is a lipid mediator that plays a crucial role in regulating various physiological processes, including tissue regeneration, immune responses, angiogenesis, and cellular signaling pathways. Previous studies have reported discordant hypotheses regarding the role of PGE2 on cancer cell growth, apoptosis, and invasion [44–48]. It has been reported that bladder cancer shows increased levels of PGE2 and decreased levels of 15‐hydroxyprostaglandin dehydrogenase, the enzyme responsible for the degradation of PGE2 [49–51]. Unlike these findings, we detected downregulation of PGE2 in patients compared to healthy controls. We believe that the lower levels of PGE2 in bladder cancer may be due to a combination of factors, including metabolic adaptations and external influences such as treatments and lifestyle factors. Understanding these mechanisms requires further investigation to elucidate the distinct biological characteristics of bladder cancer. Nonetheless, PGE2 has been found to play a critical role in promoting various hallmarks of cancer, including cell proliferation, angiogenesis, metastasis, and resistance to apoptosis.

Elucidating the roles of steroid hormones and arachidonic acid metabolism in bladder cancer biology may provide critical insights into the molecular mechanisms underlying the disease. Such knowledge could facilitate the development of novel therapeutic strategies targeting these pathways, with the potential to improve patient outcomes. Biomarkers derived from these metabolic pathways, including PGE2 and 11‐hydroxyandrosterone, show promise as diagnostic markers for early detection and monitoring of recurrence. Their quantification in biological samples may enhance the management and treatment of bladder cancer. Advancing the understanding of these metabolic pathways and their contributions to bladder cancer is likely to influence future research and clinical practice, offering new opportunities for diagnosis, prognosis, and targeted therapy. This study has some limitations, including its single‐center design, a relatively small cohort size, and the absence of longitudinal validation. Future multi‐center studies with larger cohorts and integration of other omics data are necessary to confirm and expand upon these findings. In addition, the relatively small and imbalanced control cohort may reduce statistical power and increase susceptibility to overfitting, potentially limiting the generalizability of the findings.

## 5. Conclusion

This study identified unique urinary metabolomic signatures that distinguish bladder cancer patients from healthy controls and provide insights into recurrence risk. Several altered metabolites and pathways observed in the analysis are consistent with previous bladder cancer metabolomics studies, which supports the biological relevance of these findings. Increased levels of (±)12‐HETE, 21‐hydroxypregnenolone, and 5α‐dihydrodeoxycorticosterone, together with decreased levels of 11‐hydroxyandrosterone, hippuric acid, and PGE2, highlight the involvement of arachidonic acid metabolism, steroid hormone signaling, and microbiota–host co‐metabolism in bladder cancer biology. Dysregulation of arachidonic acid metabolism has been previously reported in bladder cancer, including increased HETE species such as 5‐HETE and 15‐HETE in muscle‐invasive bladder cancer tissue [52]. While (±)12‐HETE has not been reported as a urinary biomarker for bladder cancer, its presence in the arachidonic acid metabolic pathway suggests that this observation reflects a related inflammatory lipid signature detectable in urine.

Decreased urinary levels of hippuric acid in the bladder cancer cohort are consistent with previous metabolomics studies reporting reduced hippuric acid in bladder cancer urine compared with controls [53, 54], which aligns with perturbations in microbiota–host co‐metabolism. Among the identified metabolites, 11‐hydroxyandrosterone showed the strongest predictive power for recurrence, highlighting its potential as a noninvasive biomarker for disease monitoring. Notably, several steroid hormone–related metabolites identified in this study, including 21‐hydroxypregnenolone, 5α‐dihydrodeoxycorticosterone, and 11‐hydroxyandrosterone, have not previously been reported as specific urinary biomarkers for bladder cancer. Although alterations in androgen and steroid hormone pathways, including 5α‐reductase–related metabolism, have been described in bladder cancer [55], these findings extend current knowledge by identifying distinct urinary steroid intermediates as novel candidate biomarkers. Microbiota‐derived aromatic metabolites have been implicated in bladder cancer, and p‐cresol glucuronide has been validated as a diagnostic biomarker for bladder cancer and as a staging biomarker for nonmuscle‐invasive disease [56]. In contrast, elevated urinary p‐cresol sulfate levels observed in recurrence‐positive patients in this study have not been commonly reported in prior bladder cancer metabolomics literature, indicating a novel association that may reflect recurrence‐specific microbiome–tumor interactions. Overall, these findings suggest that disrupted lipid and steroid hormone pathways, together with changes in microbiome‐associated metabolites, are central to bladder cancer development and recurrence. These results enhance understanding of bladder cancer metabolism and identify promising candidate biomarkers for early detection, prognosis, and personalized patient management. Further research with larger, multicenter cohorts and functional validation is required to confirm these associations and explore their therapeutic potential.

## Funding

No funding was received for this manuscript.

## Disclosure

An earlier version of this work was presented as an oral presentation and published as an abstract in The Turkish Journal of Biochemistry [49(S1):S105, 2024]. Available at: https://www.doi.org/10.1515/tjb-2024-49s105.

## Conflicts of Interest

The authors declare no conflicts of interest.

## Data Availability

The data that support the findings of this study are available on request from the corresponding author. The data are not publicly available due to privacy or ethical restrictions.

## References

[1] Bray F. , Laversanne M. , Sung H. et al., Global Cancer Statistics 2022: GLOBOCAN Estimates of Incidence and Mortality Worldwide for 36 Cancers in 185 Countries, CA: A Cancer Journal for Clinicians. (2024) 74, no. 3, 229–263, 10.3322/caac.21834. 38572751

[2] Saginala K. , Barsouk A. , Aluru J. S. , Rawla P. , and Padala S. A. , Epidemiology of Bladder Cancer, Medical Science. (2020) 8, no. 1, 10.3390/medsci8010015. PMC7151633 32183076

[3] Lenis A. T. , Lec P. M. , Chamie K. , and Mshs M. , Bladder Cancer: A Review, JAMA. (2020) 324, no. 19, 1980–1991, 10.1001/jama.2020.17598. 33201207

[4] Zhu C. Z. , Ting H. N. , Ng K. H. , and Ong T. A. , A Review on the Accuracy of Bladder Cancer Detection Methods, Journal of Cancer. (2019) 10, no. 17, 4038–4044, 10.7150/jca.28989, 2-s2.0-85070483562. 31417648 PMC6692607

[5] Yang Z. , Song F. , and Zhong J. , Urinary Biomarkers in Bladder Cancer: Fda-Approved Tests and Emerging Tools for Diagnosis and Surveillance, Cancers (Basel). (2025) 17, no. 21, 10.3390/cancers17213425. PMC12607409 41228219

[6] Kata S. G. , Zreik A. , Ahmad S. , Chłosta P. , and Aboumarzouk O. , Concurrent Bladder Cancer in Patients Undergoing Photodynamic Diagnostic Ureterorenoscopy: How Many Lesions Do We Miss Under White Light Cystoscopy?, Central European Journal of Urology. (2016) 69, no. 4, 334–340, 10.5173/ceju.2016.896, 2-s2.0-85019644467. 28127447 PMC5260463

[7] Ma J. , Roumiguie M. , Hayashi T. et al., Long-Term Recurrence Rates of Low-Risk Non-Muscle-Invasive Bladder Cancer-How Long is Cystoscopic Surveillance Necessary?, European Urology Focus. (2024) 10, no. 1, 189–196, 10.1016/j.euf.2023.06.012. 37442722

[8] Maas M. , Todenhöfer T. , and Black P. C. , Urine Biomarkers in Bladder Cancer—Current Status and Future Perspectives, Nature Reviews Urology. (2023) 20, no. 10, 597–614, 10.1038/s41585-023-00773-8. 37225864

[9] Ahangar M. , Mahjoubi F. , and Mowla S. J. , Bladder Cancer Biomarkers: Current Approaches and Future Directions, Frontiers in Oncology. (2024) 14, 10.3389/fonc.2024.1453278. PMC11638051 39678505

[10] Nizioł J. , Ossoliński K. , Płaza-Altamer A. et al., Untargeted Ultra-High-Resolution Mass Spectrometry Metabolomic Profiling of Blood Serum in Bladder Cancer, Scientific Reports. (2022) 12, no. 1, 10.1038/s41598-022-19576-9. PMC9452537 36071106

[11] di Meo N. A. , Loizzo D. , Pandolfo S. D. et al., Metabolomic Approaches for Detection and Identification of Biomarkers and Altered Pathways in Bladder Cancer, International Journal of Molecular Sciences. (2022) 23, no. 8, 10.3390/ijms23084173. PMC9030452 35456991

[12] Tan G. , Wang H. , Yuan J. et al., Three Serum Metabolite Signatures for Diagnosing Low-Grade and High-Grade Bladder Cancer, Scientific Reports. (2017) 7, no. 1, 10.1038/srep46176, 2-s2.0-85017096898. PMC5382774 28382976

[13] Doğan H. O. , Metabolomics: A Review of Liquid Chromatography Mass Spectrometry-Based Methods and Clinical Applications, Turkish Journal of Biochemistry. (2024) 49, no. 1.

[14] Wang Z. , Liu X. , Sun H. et al., UPLC-MS Based Urine Untargeted Metabolomic Analyses to Differentiate Bladder Cancer From Renal Cell Carcinoma, BMC Cancer. (2019) 19, no. 1, 10.1186/s12885-019-6354-1. PMC6896793 31805976

[15] Ossoliński K. , Ruman T. , Copié V. et al., Targeted and Untargeted Urinary Metabolic Profiling of Bladder Cancer, Journal of Pharmacy Biomedicine Analytical. (2023) 233, 10.1016/j.jpba.2023.115473. 37229797

[16] Sumner L. W. , Amberg A. , Barrett D. et al., Proposed Minimum Reporting Standards for Chemical Analysis Chemical Analysis Working Group (CAWG) Metabolomics Standards Initiative (MSI), Metabolomics. (2007) 3, no. 3, 211–221, 10.1007/s11306-007-0082-2, 2-s2.0-34748888866. 24039616 PMC3772505

[17] Contursi A. , Schiavone S. , Dovizio M. et al., Platelets Induce Free and Phospholipid-Esterified 12-Hydroxyeicosatetraenoic Acid Generation in Colon Cancer Cells by Delivering 12-Lipoxygenase, Journal of Lipid Research. (2021) 62, 10.1016/j.jlr.2021.100109. PMC8456051 34428433

[18] Laquer V. , Dellinger R. W. , Mannering I. et al., 12-Hydroxyeicosatetraenoic Acid Levels are Increased in Actinic Keratoses and Squamous Cell Carcinoma, Journal of the American Academy of Dermatology. (2018) 79, no. 6, 1152–1153, 10.1016/j.jaad.2018.05.1251, 2-s2.0-85055046523. 29902547 PMC6260832

[19] Moreno J. J. , New Aspects of the Role of Hydroxyeicosatetraenoic Acids in Cell Growth and Cancer Development, Biochemical Pharmacology. (2009) 77, no. 1, 1–10, 10.1016/j.bcp.2008.07.033, 2-s2.0-56649118965. 18761324

[20] Zhang C. , Ma C. , Yao H. et al., 12-Lipoxygenase and 12-Hydroxyeicosatetraenoic Acid Regulate Hypoxic Angiogenesis and Survival of Pulmonary Artery Endothelial Cells via PI3K/Akt Pathway, American Journal of Physiology-Lung Cellular and Molecular Physiology. (2018) 314, no. 4, L606–L616, 10.1152/ajplung.00049.2017, 2-s2.0-85045523929. 29074487

[21] Honn K. V. , Tang D. G. , Gao X. et al., 12-Lipoxygenases and 12 (S)-HETE: Role in Cancer Metastasis, Cancer and Metastasis Reviews. (1994) 13, no. 3-4, 365–396, 10.1007/bf00666105, 2-s2.0-0028607279. 7712597

[22] Liu Q. , Tan W. , Che J. et al., 12-HETE Facilitates Cell Survival by Activating the Integrin-Linked Kinase/NF-κB Pathway in Ovarian Cancer, Cancer Management and Research. (2018) 10, 5825–5838, 10.2147/cmar.s180334, 2-s2.0-85057622027. 30510451 PMC6248369

[23] Dowling P. , Henry M. , Meleady P. et al., Metabolomic and Proteomic Analysis of Breast Cancer Patient Samples Suggests That Glutamate and 12-HETE in Combination With CA15-3 May Be Useful Biomarkers Reflecting Tumour Burden, Metabolomics. (2015) 11, no. 3, 620–635, 10.1007/s11306-014-0723-1, 2-s2.0-84929464709.

[24] Madhunapantula S. V. , Mosca P. , and Robertson G. P. , Steroid Hormones Drive Cancer Development, Cancer Biology & Therapy. (2010) 10, no. 8, 765–766, 10.4161/cbt.10.8.13531, 2-s2.0-78049288555. 20935464

[25] Uemura M. , Honma S. , Chung S. et al., 5αDH‐DOC (5Α‐Dihydro‐Deoxycorticosterone) Activates Androgen Receptor in Castration‐Resistant Prostate Cancer, Cancer Science. (2010) 101, no. 8, 1897–1904, 10.1111/j.1349-7006.2010.01620.x, 2-s2.0-77954847434. 20560974 PMC11158608

[26] Birtle A. , Freeman A. , and Munson P. , The Androgen Receptor Revisited in Urothelial Carcinoma, Histopathology. (2004) 45, no. 1, 98–99, 10.1111/j.1365-2559.2004.01841.x, 2-s2.0-3242781592. 15228457

[27] Ide H. , Inoue S. , and Miyamoto H. , Histopathological and Prognostic Significance of the Expression of Sex Hormone Receptors in Bladder Cancer: A Meta-Analysis of Immunohistochemical Studies, PLoS One. (2017) 12, no. 3, 10.1371/journal.pone.0174746, 2-s2.0-85016603223. PMC5375178 28362839

[28] Ikuta K. , Ejima A. , Abe S. , and Shimba A. , Control of Immunity and Allergy by Steroid Hormones, Allergology International. (2022) 71, no. 4, 432–436, 10.1016/j.alit.2022.07.006. 35973911

[29] Alonso-Diez Á. , Cáceres S. , Peña L. , Crespo B. , and Illera J. C. , Anti-Angiogenic Treatments Interact With Steroid Secretion in Inflammatory Breast Cancer Triple Negative Cell Lines, Cancers (Basel). (2021) 13, no. 15, 10.3390/cancers13153668. PMC8345132 34359570

[30] Chakraborty S. , Pramanik J. , and Mahata B. , Revisiting Steroidogenesis and Its Role in Immune Regulation With the Advanced Tools and Technologies, Genes and Immunity. (2021) 22, no. 3, 125–140, 10.1038/s41435-021-00139-3. 34127827 PMC8277576

[31] Pallister T. , Jackson M. A. , Martin T. C. et al., Hippurate as a Metabolomic Marker of Gut Microbiome Diversity: Modulation by Diet and Relationship to Metabolic Syndrome, Scientific Reports. (2017) 7, no. 1, 10.1038/s41598-017-13722-4, 2-s2.0-85032017355. PMC5651863 29057986

[32] Pruss K. M. , Chen H. , Liu Y. et al., Host-Microbe Co-Metabolism via MCAD Generates Circulating Metabolites Including Hippuric Acid, Nature Communications. (2023) 14, no. 1, 10.1038/s41467-023-36138-3. PMC9889317 36720857

[33] Lees H. J. , Swann J. R. , Wilson I. D. , Nicholson J. K. , and Holmes E. , Hippurate: The Natural History of a Mammalian-Microbial Cometabolite, Journal of Proteome Research. (2013) 12, no. 4, 1527–1546, 10.1021/pr300900b, 2-s2.0-84875919841. 23342949

[34] Brial F. , Chilloux J. , Nielsen T. et al., Human and Preclinical Studies of the Host-Gut Microbiome co-Metabolite Hippurate as a Marker and Mediator of Metabolic Health, Gut. (2021) 70, no. 11, 2105–2114, 10.1136/gutjnl-2020-323314. 33975870 PMC8515120

[35] Gryp T. , Vanholder R. , Vaneechoutte M. , and Glorieux G. , p-Cresyl Sulfate, Toxins. (2017) 9, no. 2, 10.3390/toxins9020052, 2-s2.0-85011629242. PMC5331431 28146081

[36] Watanabe H. , Miyamoto Y. , Honda D. et al., p-Cresyl Sulfate Causes Renal Tubular Cell Damage by Inducing Oxidative Stress by Activation of NADPH Oxidase, Kidney International. (2013) 83, no. 4, 582–592, 10.1038/ki.2012.448, 2-s2.0-84875710102. 23325087

[37] Shiba T. , Kawakami K. , Sasaki T. et al., Effects of Intestinal Bacteria-Derived p-Cresyl Sulfate on Th1-type Immune Response In Vivo and In Vitro, Toxicology and Applied Pharmacology. (2014) 274, no. 2, 191–199, 10.1016/j.taap.2013.10.016, 2-s2.0-84890875505. 24161588

[38] Mutsaers H. A. , Caetano-Pinto P. , Seegers A. E. et al., Proximal Tubular Efflux Transporters Involved in Renal Excretion of p-Cresyl Sulfate and p-Cresyl Glucuronide: Implications for Chronic Kidney Disease Pathophysiology, Toxicology in Vitro. (2015) 29, no. 7, 1868–1877, 10.1016/j.tiv.2015.07.020, 2-s2.0-84938780478. 26216510

[39] Wu T. K. , Wei C. W. , Pan Y. R. , Hsu R. J. , Wu C. Y. , and Yu Y. L. , The Uremic Toxin p-Cresyl Sulfate Induces Proliferation and Migration of Clear Cell Renal Cell Carcinoma via microRNA-21/HIF-1α Axis Signals, Scientific Reports. (2019) 9, no. 1, 10.1038/s41598-019-39646-9, 2-s2.0-85062280096. PMC6397167 30824757

[40] Marques J. G. , Shokry E. , Frivolt K. et al., Metabolomic Signatures in Pediatric Crohn’s Disease Patients With Mild or Quiescent Disease Treated With Partial Enteral Nutrition: A Feasibility Study, SLAS Technology. (2021) 26, no. 2, 165–177, 10.1177/2472630320969147. 33207993 PMC7985853

[41] González-Domínguez R. , Jáuregui O. , Queipo-Ortuño M. I. , and Andrés-Lacueva C. , Characterization of the Human Exposome by a Comprehensive and Quantitative Large-Scale Multianalyte Metabolomics Platform, Analytical Chemistry. (2020) 92, no. 20, 13767–13775, 10.1021/acs.analchem.0c02008. 32966057

[42] Ganguly S. , Naik D. , Muskara A. , and Mian O. Y. , The Nexus of Endocrine Signaling and Cancer: How Steroid Hormones Influence Genomic Stability, Endocrinology. (2021) 162, no. 1, 10.1210/endocr/bqaa177. PMC7707372 33260197

[43] Anderson A. C. and Acharya N. , Steroid Hormone Regulation of Immune Responses in Cancer, Immunometabolism. (2022) 4, no. 4, 10.1097/in9.0000000000000012. PMC9622373 36337733

[44] Santiso A. , Heinemann A. , and Kargl J. , Prostaglandin E2 in the Tumor Microenvironment, A Convoluted Affair Mediated by EP Receptors 2 and 4, Pharmacological Reviews. (2024) 76, no. 3, 388–413, 10.1124/pharmrev.123.000901. 38697857

[45] Greenhough A. , Smartt H. J. , Moore A. E. et al., The COX-2/PGE 2 Pathway: Key Roles in the Hallmarks of Cancer and Adaptation to the Tumour Microenvironment, Carcinogenesis. (2009) 30, no. 3, 377–386, 10.1093/carcin/bgp014, 2-s2.0-62349138124. 19136477

[46] Huang S. K. , White E. S. , Wettlaufer S. H. et al., Prostaglandin E2 Induces Fibroblast Apoptosis by Modulating Multiple Survival Pathways, The FASEB Journal. (2009) 23, no. 12, 4317–4326, 10.1096/fj.08-128801, 2-s2.0-72749127813. 19671668 PMC2812040

[47] Lalier L. , Pedelaborde F. , Braud C. , Menanteau J. , M Vallette F. , and Olivier C. , Increase in Intracellular PGE 2 Induces Apoptosis in Bax-Expressing Colon Cancer Cell, BMC Cancer. (2011) 11, 1–9, 10.1186/1471-2407-11-153, 2-s2.0-79955157821. 21524287 PMC3097003

[48] Fard S. S. , Tehrani M. J. , and Ardekani A. M. , Prostaglandin E2 Induces Growth Inhibition, Apoptosis and Differentiation in T and B Cell-Derived Acute Lymphoblastic Leukemia Cell Lines (CCRF-CEM and Nalm-6), Prostaglandins Leukotrienes and Essential Fatty Acids. (2012) 87, no. 1, 17–24, 10.1016/j.plefa.2012.04.012, 2-s2.0-84864113055. 22749740

[49] Tseng-Rogenski S. , Gee J. , Ignatoski K. W. et al., Loss of 15-Hydroxyprostaglandin Dehydrogenase Expression Contributes to Bladder Cancer Progression, The American Journal of Pathology. (2010) 176, no. 3, 1462–1468, 10.2353/ajpath.2010.090875, 2-s2.0-77749245858. 20093479 PMC2832165

[50] Tseng-Rogenski S. S. and Liebert M. , 15-Hydroxyprostagladin Dehydrogenase (PGDH) is a Potential Bladder Cancer Tumor Suppressor, American Association for Cancer Research. (2006) .

[51] Woolbright B. L. , Pilbeam C. C. , and Taylor J. A.III, Prostaglandin E2 as a Therapeutic Target in Bladder Cancer: From Basic Science to Clinical Trials, Prostaglandins & Other Lipid Mediators. (2020) 148, 10.1016/j.prostaglandins.2020.106409. 31931078

[52] Sahu D. , Lotan Y. , Wittmann B. , Neri B. , and Hansel D. E. , Metabolomics Analysis Reveals Distinct Profiles of Nonmuscle-Invasive and Muscle-Invasive Bladder Cancer, Cancer Medicine. (2017) 6, no. 9, 2106–2120, 10.1002/cam4.1109, 2-s2.0-85026660980. 28766915 PMC5603845

[53] Loras A. , Martínez-Bisbal M. C. , Quintás G. , Gil S. , Martínez-Máñez R. , and Ruiz-Cerdá J. L. , Urinary Metabolic Signatures Detect Recurrences in Non-Muscle Invasive Bladder Cancer, Cancers (Basel). (2019) 11, no. 7, 10.3390/cancers11070914, 2-s2.0-85068701241. PMC6678457 31261883

[54] Loras A. , Trassierra M. , Sanjuan-Herráez D. et al., Bladder Cancer Recurrence Surveillance by Urine Metabolomics Analysis, Scientific Reports. (2018) 8, no. 1, 10.1038/s41598-018-27538-3, 2-s2.0-85048707242. PMC6004013 29907864

[55] Amara C. S. , Vantaku V. , Lotan Y. , and Putluri N. , Recent Advances in the Metabolomic Study of Bladder Cancer, Expert Review of Proteomics. (2019) 16, no. 4, 315–324, 10.1080/14789450.2019.1583105, 2-s2.0-85063535734. 30773067 PMC6538267

[56] Oto J. , Fernández-Pardo Á. , Roca M. et al., LC-MS Metabolomics of Urine Reveals Distinct Profiles for Non-Muscle-Invasive and Muscle-Invasive Bladder Cancer, World Journal of Urology. (2022) 40, no. 10, 2387–2398, 10.1007/s00345-022-04136-7. 36057894

