# Correction to “Metabolic Profiling Reveals Insights Into Bladder Cancer Pathogenesis and Recurrence”

**DOI:** 10.1155/aiu/9810919

**Published:** 2026-05-27

H. Saygın, S. Bolat, D. Kablan, et al., “Metabolic Profiling Reveals Insights Into Bladder Cancer Pathogenesis and Recurrence,” *Advances in Urology*, 20266524384, https://doi.org/10.1155/aiu/6524384.

In the article, there was an error in the name of author Meltem Kurt Yenihan, which was previously stated as Meltem Kurt. This has now been corrected in the article.

We apologize for this error.

